# Directional, super‐hydrophobic cellulose nanofiber/polyvinyl alcohol/montmorillonite aerogels as green absorbents for oil/water separation

**DOI:** 10.1049/nbt2.12008

**Published:** 2021-02-14

**Authors:** Nannan Rong, Zhaoyang Xu, Shengcheng Zhai, Lijie Zhou, JiaJia Li

**Affiliations:** ^1^ College of Materials Science and Engineering Nanjing Forestry University Nanjing China; ^2^ Key Laboratory of Bio‐based Material Science & Technology Northeast Forestry University Harbin China; ^3^ Jiangsu Co‐Innovation Center of Efficient Processing and Utilization of Forest Resources Nanjing Forestry University Nanjing China

## Abstract

Nowadays, the problem of oil spill and organic solvent pollution has become more and more serious, and developing a green and efficient treatment method has become a research hotspot. Herein, the preparation of porous super‐hydrophobic aerogel by directional freezing with cellulose nanofibre (CNF) as the base material, polyvinyl alcohol (PVA) as the cross‐linking agent and montmorillonite (MMT) as the modifier and filler, followed by hydrophobic treatment with chemical vapour deposition is reported. The prepared composite aerogel presented three‐dimensional inter‐perforation network structure, low density (26.52 mg⋅cm^−3^), high porosity (96.1 %) and good hydrophobicity (water contact angle of 140°). Notably, the composite aerogel has a good adsorption effect on different oils and organic solutions, and its adsorption capacity can reach 40–68 times of its initial weight. After complete adsorption, the aerogel could be easily collected. More importantly, the composite aerogel had high strength, whose compressive stress at 70 % strain reached 0.15 MPa and could bear over 1290 times its weight without deformation after 2 weeks. A new, green, simple and efficient absorbent for the adsorption of oils and organic solvents is provided.

## 1 INTRODUCTION

As is known to all, the geographical location of different countries in the world, the categories and reserves of fossil resources stored are different. Therefore, fossil energy need to be transported to each other, and in the existing transportation methods, sea transportation accounts for a large proportion. Once an accident occurs, it will trigger a series of environmental problems. Historically, a series of disasters caused by improper exploitation and transportation of oil are common occurrences, such as the Exxon Valdez incident in 1989, and the Gulf of Mexico accident in 2010, which was the largest accidental marine oil spill [1,2]. These accidents not only have disastrous consequences on the ecological environment, seriously threaten the survival of people, but also need to pay a lot of to deal with them, or even difficult to solve [3, 4, 5]. For oil spill accident treatment, there are two major methods: i) chemical method, including combustion in situ and dispersant precipitate degradation and ii) physical method, mainly mechanical salvage and recovery and adsorption of adsorbent [3, 4, 5]. Among above approaches, the adsorbent is considered to be the most effective, convenient and environmentally friendly, with the absorbed oil can be extracted from the adsorbent for use, or directly incinerated in situ without secondary pollution [3, 4, 5]. At present, many materials have been used to prepare adsorbents, mainly including mineral materials, carbon‐based materials, polymer‐based materials and cellulosic materials, but all of have some shortcomings. For example, mineral materials are hard to collect after adsorption and the selectivity of oil and water is poor. The production process of carbon‐based materials is complex and expensive. The polymer‐based materials are hard to degrade naturally, and the adsorption effect of natural‐based materials is worse. Therefore, it is necessary to research a new type of material with affordable, high strength, sustainable and high adsorption capacity. As everyone knows, aerogel has the characteristics of low density, large specific surface area, high porosity and good adsorption property. It fully meets the above requirements and is one of the ideal materials for oil/organic adsorbents [8, 9, 10, 11, 12].

Cellulose is not only the most abundant natural polymer material on Earth, but also the largest renewable resource in storage. More important, because of the properties of cellulose nanofibre (CNF)s, such as environmental friendliness, biocompatibility and biodegradability, it has become a research focus in the field of materials and is regarded as open up a new way for the development of materials, and has been widely used in medicine [13,14], environment [9, 10, 11, 12] and other fields. In industrial production, CNF can be simply prepared using the simplest raw materials through physical or chemical methods [15]. According to the above‐mentioned methods, preparing CNF, and then the aerogel or sponge prepared by CNF will form a three‐dimensional network pore structure, which has the properties of ultra‐low density and large specific surface area. Therefore, it can be regarded as one of the best choices for adsorbing materials [16,17]. However, the aerogel prepared from CNF still have some shortcomings, such as low strength, poor reusability and adsorption efficiency, which seriously affect their application [18, 19, 20]. Besides, CNF are both hydrophilic and oleophilic, so it is necessary to be modified as an oil‐absorbing material.

Polyvinyl alcohol (PVA) is a kind of water‐soluble, environment‐friendly and biocompatible degradable material. PVA has a wide range of application in many fields [21, 22, 23]. Because of the presence of many hydroxyl groups on the PVA molecular chain, PVA‐modified cellulosic membranes [24, 25, 26], aerogels [27, 28, 29] and other composite materials [30,31] have great advantages compared with their raw materials, which not only enhance their original performance, but also give them no previous characteristics. Also, some scholars have confirmed that when the materials are modified, PVA can improve the degree of silane modification of the material and make the hydrophobicity better [32].

Montmorillonite, also known as microcrystalline kaolinite, is a natural mineral of silicate. The molecular structure formula is M_+*y*_ (Al_*y*_, Mg_2‐*y*_) (Si_4_) O_10_ (OH)_2_·*n* H_2_O, and the microstructure is a lamellar structure consisting mainly of one Al_2_O_3_ octahedron sandwiched in the middle of two SiO_2_ tetrahedrons. Because montmorillonite has a unique lamellar nanostructure, inter‐layer reactivity can be designed, with a large specific surface area and the thickness–diameter ratio [33]. When montmorillonite is compounded with other materials, whose special structure will greatly improve the mechanical properties, transmittance and thermal properties of the composite materials [34,35]. Therefore, MMT has been widely used in flame retardant, polymer enhancement, adsorption and other fields [36, 37, 38, 39]. Although researchers have done a lot of experiments in the preparation and application of montmorillonite composites materials, most of them only focus on the flame retardant and mechanical properties of montmorillonite, and relatively little research on oil absorption performance, not to mention the preparation of gel materials by cellulose and montmorillonite [40].

We report a new, simple and green method, which is to use liquid nitrogen to conduct directional freezing of CNF/PVA/montmorillonite composite aerogel. During freezing, the aerogel will form a directional pore structure, giving the aerogels three‐dimensional porousness, ultra‐low density, high porosity and the addition of montmorillonite will enhance the aerogels' various properties, such as mechanical properties and adsorption properties. Compared with pure cellulose aerogel and non‐direction aerogel, their mechanical properties and adsorption properties were significantly enhanced. We did a preliminary experiment before deciding on the best ratio. The composite aerogel with CNF, PVA and MMT mass ratio of 1:1:1 was prepared by directional freeze‐drying, which has the best performance. Then, aerogels were hydrophobically modified by simple chemical vapour deposition with methyltrimethoxysilane to obtain a hydrophobic CNF/PVA/MMT composite aerogel (MCPMA). The aerogel exhibited excellent oil–water selectivity, oil absorption properties and high mechanical strength. After the adsorption was completed, the aerogels can be recycled simply.

## 2 MATERIALS AND METHOD

### 2.1 Materials

Bamboo powder (60 mesh) was purchased from Chongqing Dongyi Xiabu Co, Ltd., (Chongqing, China) and was the raw material for preparing CNF. Montmorillonite was provided by Shenzhen Mingsen plastic materials Co, Ltd., (Shenzhen, China). PVA type 1799 (Degree of alcoholysis: 98–99% mol⋅mol^−1^), potassium hydroxide (KOH), Sudan III, Methyltrimethoxysilane (MTMS) (98.0 wt%), acetic acid (CH_3_COOH), hydrochloric acid (HCl), sodium chlorite (NaClO_2_), benzene and ethanol. All materials are analytically pure, need no further purification and have not been used before. All the water used in the whole experiment was deionized water prepared in our laboratory.

### 2.2 Preparation of cellulose nanofibres

The treatment method of bamboo powder and the preparation of CNF has been mentioned in the previous literature [41]. The final CNF suspension concentration was 2.4 wt% was stored in the refrigerator at 4°C for future utilisation.

### 2.3 Preparation of CNF/PVA/MMT composite aerogel (CPMA)

**FIGURE 1:**
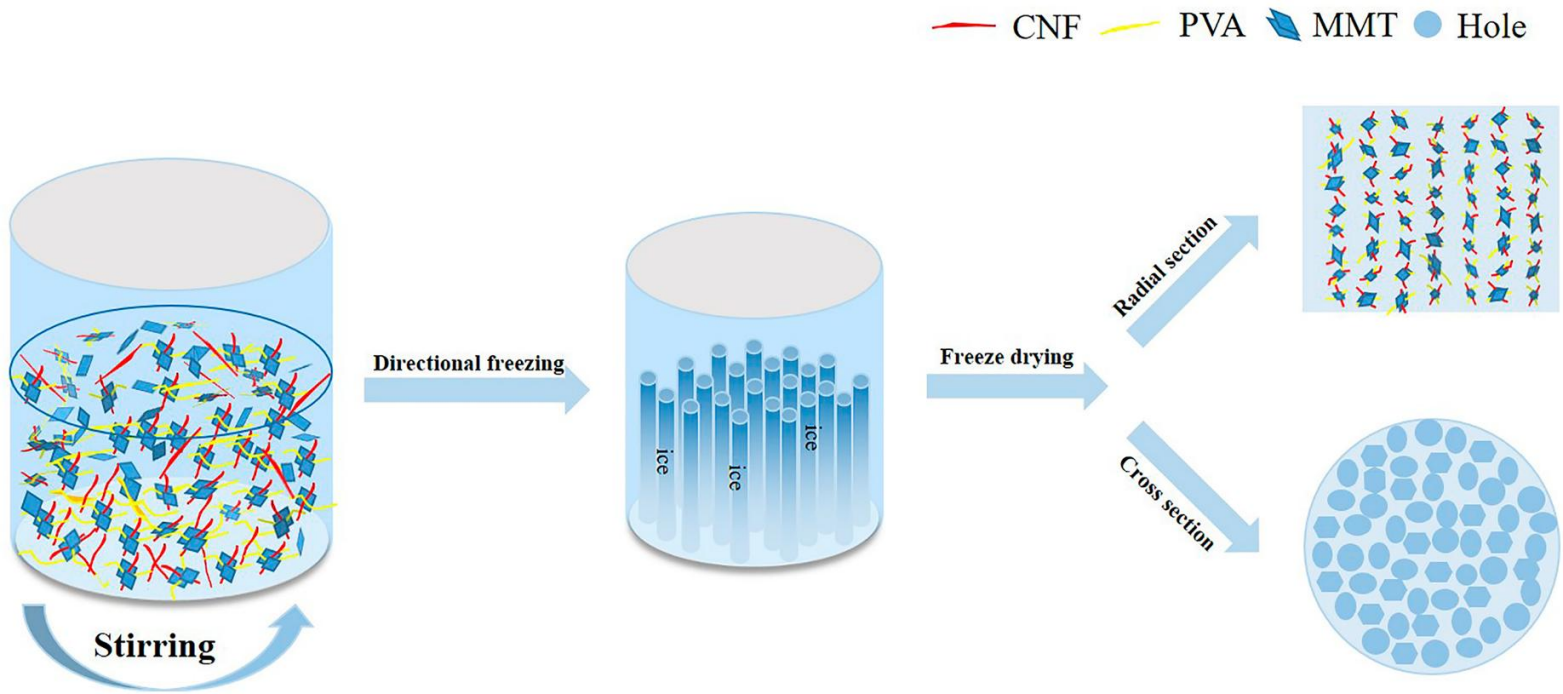
Preparation of CNF/PVA/MMT aerogel by directional method

The total mass of the mixed solution was 40 g, the CNF concentration was set at 0.6 wt%, and CNF, PVA and MMT were added in a certain proportion. Take CNF:PVA:MMT = 1:1:1 as an example. First, 10 g CNF suspension with a concentration of 2.4 wt% was prepared and ultrasound for 0.5 h. Then 0.24 g MMT was dissolved in 9.76 ml deionized water and ultrasound 0.5 h. After that, the CNF suspension was mixed with the MMT suspension and ultrasound again for 0.5 h to disperse the mixture evenly. At the same time, 0.24 g PVA was dissolved in 19.76 ml deionized water, kept in the oil bath at 60°C for 0.5 h, then add the mixture into the PVA solution, heated to 95°C and stirred for 3 h. The liquid mixture was then poured in a special circular mould, and then pre‐cooled at 4°C overnight in the refrigerator to avoid macroscopic cracking during freeze‐casting. After later, the pre‐cooled samples were placed in special glassware, and the liquid nitrogen was used for directional freezing. They were then freeze‐dried in a FreeZone 6 L benchtop freeze‐drying system equipped with a bulk tray dryer (Labconco Corporation, United States) at a sublimating temperature of −53°C and a pressure of 0.021 mbar for 3 days. Finally, in the freeze dryer, the directional growth of ice crystals was gradually sublimated, and aerogels form a directional pore structure (Figure 1). All aerogels did not show cracks, and the volumetric shrinkage rate was less than 6.0%. The proportion of aerogels is shown in Table 1.

**TABLE 1:** Example table and raw material ratio

Samples	CNF/g	PVA/g	MMT/g	Total/g
CNF	10	0	0	40
CNF:PVA:MMT	10	0.24	0.12	40
CNF:PVA:MMT	10	0.24	0.24	40
CNF:PVA:MMT	10	0.24	0.48	40

### 2.4 Silanisation reaction of CNF/PVA/MMT aerogel (MCPMA)

**FIGURE 2:**
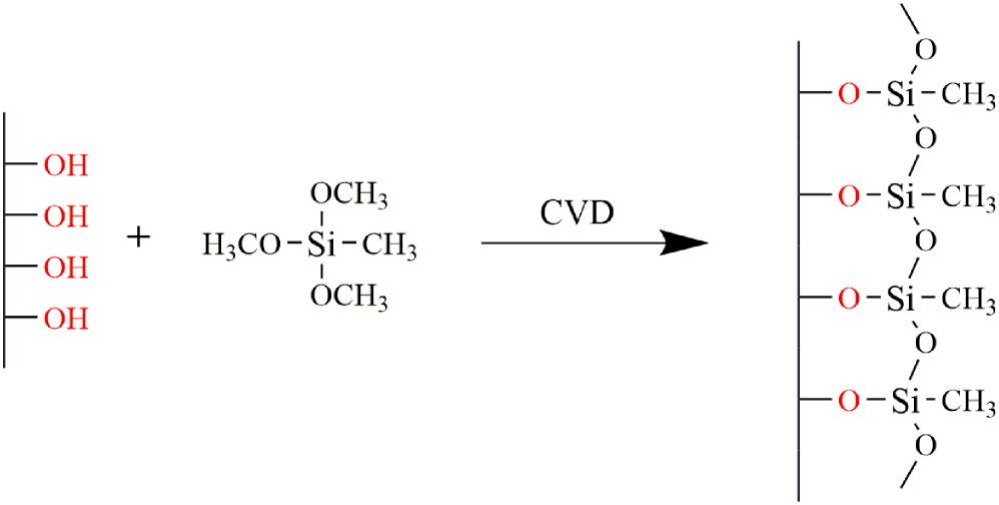
Schematic diagram of modified aerogel by methyltrimethoxysilane

With MTMS as modifier, two small glass bottles, 2 ml MTM, 1 ml H_2_O and prepared aerogel samples were placed in a 500 ml sealed container and reacted at 70°C for 2 h. After the reaction (Figure 2), the aerogel was rinsed with a mixture of ethanol and methanol to remove the unreacted silane, and then vacuum dried for 24 h at 60°C. The last, the aerogels were placed in the dryer for standby.

### 2.5 Characterisation

#### 2.5.1 Scanning electron microscopy (SEM)

First, the aerogel to be tested was dried at 60°C for 24 h. At the beginning of the test, the aerogel was placed on the cushion and made to the right size, and then sputter‐coated with a thin layer of Au. Subsequently, the as‐prepared samples were observed using the Quanta 2000 scanning electron microscope (America) at an accelerating voltage of 10 kV in secondary electron mode.

#### 2.5.2 Fourier transform infrared (FTIR) spectroscopy

FTIR spectra were recorded on a Vector 80 V infrared spectrum instrument (Bruker Corporation, Germany). The scan range was 500–4000 cm^−1^. At the same time, Smart ATR mode was selected, the interval time and resolution were 4  and ±2 cm^−1^, and each sample was scanned 32 times.

#### 2.5.3 X‐ray photoelectron spectroscopy (XPS)

The surface compositions of aerogel samples were analysed XPS (AXIS UltraDLD, England, UK). The wide sweep and narrow sweep were used for qualitative and quantitative analysis of elements, such as C, O and Si, respectively. The dwell time was 0.1 s, the monochromatic power 600 W and vacuum degree of analysis room was greater than 7 × 10^−8^ Pa.

#### 2.5.4 Mechanical property test

The compression test was carried out at room temperature using Shimadzu CMT4204 (Kyoto, Japan) Universal mechanical testing machine. The test samples were 16 mm in height and 20 mm in diameter, and the loading rate of the machine in the test was set to 5 mm⋅min^−1^.

#### 2.5.5 Water contact angle (WCA) measurement of aerogels

WCA measurements were implemented to investigate, analyse and verify the hydrophobicity of MTMS‐modified CPM aerogels. On an OCA (Data Physics Instruments) using a droplet (2 μl) of deionized water as an indicator, capture the contact angle of the droplets and the surface of the aerogel.

#### 2.5.6 Density and porosity of MCPMA

The apparent volumetric mass density of an MCPMA was calculated using the following formula:

(1) ρ=m/v

where *m* and *v* are the mass (g) and volume (cm^3^) of the aerogel, respectively.

The porosity (*P*, %) of aerogel was calculated according to Equation (2), where *ρ*_s_ was the density of solid.

(2) P(%)=(1−ρ/ρs)×100%

The density (*ρ*_s_) of the solid material was calculated according to Equation (3) based on the solid density of each component and their weight ratios:

(3) $$\rho s=1/\left(W_{1}/\rho_{1}\;+\;W_{2}/\rho_{2}\;+\;W_{3}/\rho_{3}+\;W_{4}/\rho_{4}\right)$$

where *W* (1, 2, 3, 4) and *ρ* (1, 2, 3, 4) are the weight percentages of the different components and the solid densities of CNF, PVA, MMT and MTMS, respectively. The densities of the CNF, PVA, MMT and MTMS used here were 1.470, 1.278, 1.020 and 0.955 g⋅cm^−3^, respectively.

#### 2.5.7 Oil sorption selectivity of MCPM aerogels

First, a certain amount of water and oil were weighed separately, and then the oil was dyed and mixed with water to form a distinct stratification between the oil and water. Later the aerogel was put into the mixed solution, and then the adsorbed aerogel and water were weighed. Oil sorption selectivity was defined as the ratio of the total weight of aerogel after adsorption and the total weight of aerogel and oil before adsorption.

#### 2.5.8 Absorption capacities for oils or organic solvents

In a typical absorption test, when weighing, there was no oil dripping on the surface, and it was weighted every 10 s until the weight of the aerogel has not changed. The adsorption capacity (*g*_c_/*g*_i_), defined as the weight (*g*_c_) of aerogel after the adsorption was completely divided by the initial weight (*g*_i_) of aerogel. Each adsorption test was performed at least five times, and the average of multiple times was used as the final data.

## 3 RESULT AND DISCUSSION

### 3.1 Microstructure

**FIGURE 3:**
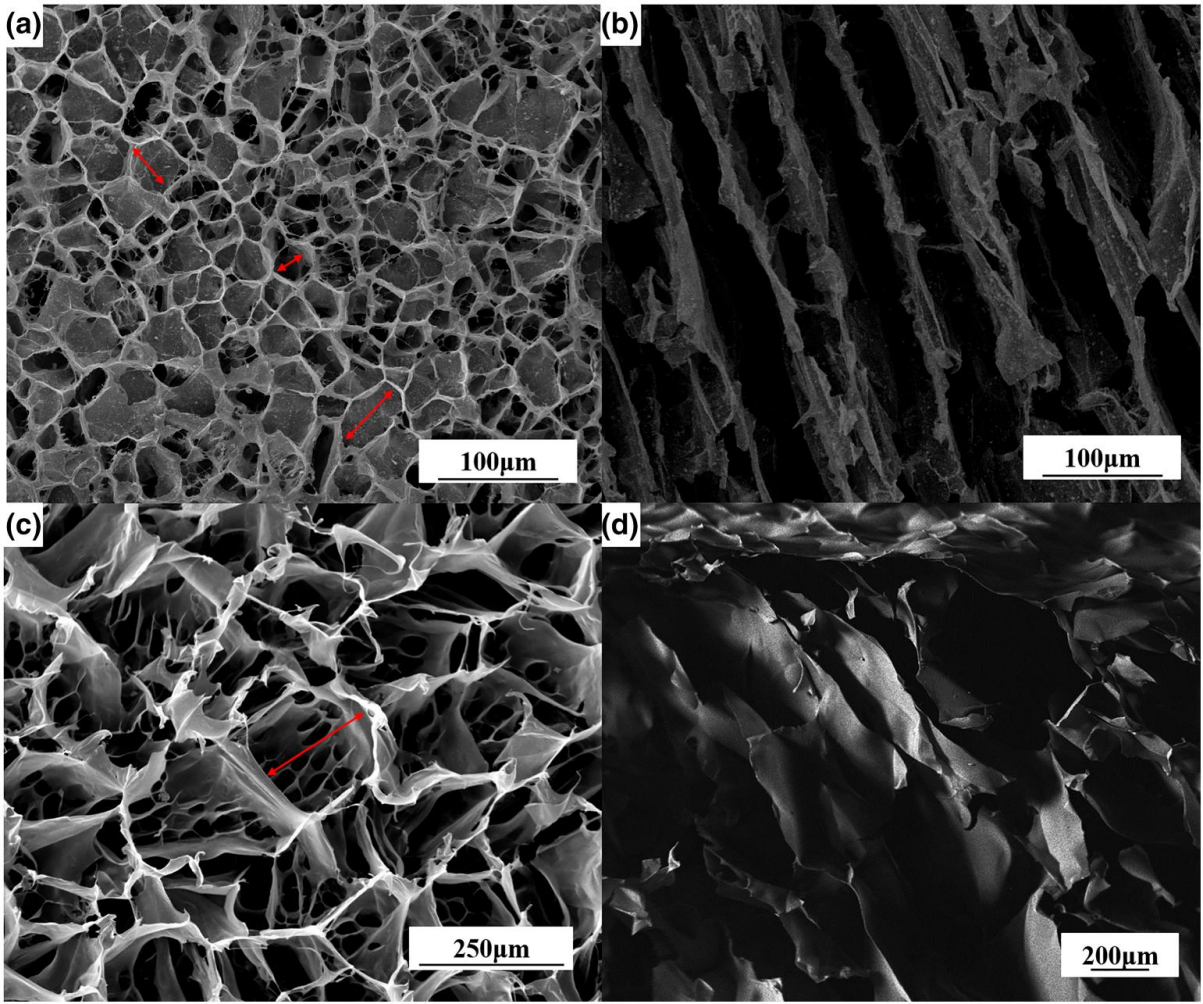
Microstructures of aerogels formed by different materials and freeze‐drying. (a) and (b) are the cross section and vertical section of CPMA by liquid nitrogen, respectively; (c) is the figures of the pure CNF aerogel cross section and (d) is the vertical section of CPMA by refrigerator

To explore the effect of montmorillonite addition on the micromorphology of aerogel and the difference of aerogel morphology after liquid nitrogen directional freezing and refrigeration freezing, we observed the microstructure by SEM (Figure 3a–3d). Previous studies have shown that the solidification mode affects the microstructure and pore structure of microporous materials [19]. Figure 3a and 3b shows the cross section and radial sections of CPMA used liquid nitrogen‐directed freezing. Figure 3c is the cross section of pure CNF aerogel, and Figure 3d is the radial section of the aerogel frozen in the refrigerator. The diameter of the hole in the pure CNF aerogel was 150–200 μm. When added montmorillonite, the pore shape of aerogel became similar to hexagonal structure, the diameter of holes were reduced to 30–50 μm and the size and distribution were more uniform (Figure 3a). Comparing Figure 3b and 3d, it could find that by directional freezing of aerogel, cellulose grows vertically and parallel inside the aerogel, created a vertically oriented structure, which enhances the mechanical properties and adsorption properties. Figure 3d, aerogel frozen in refrigerator, the size difference increases and a continuous layered structure appears, resulting in a smaller space, which was not conducive to the adsorption and temporary storage of oil and organic solvents.

### 3.2 Chemical properties

To evaluate the formation of MTMS/CNF/PVA/MMT composites, we used FTIR spectrometer and XPS.

**FIGURE 4:**
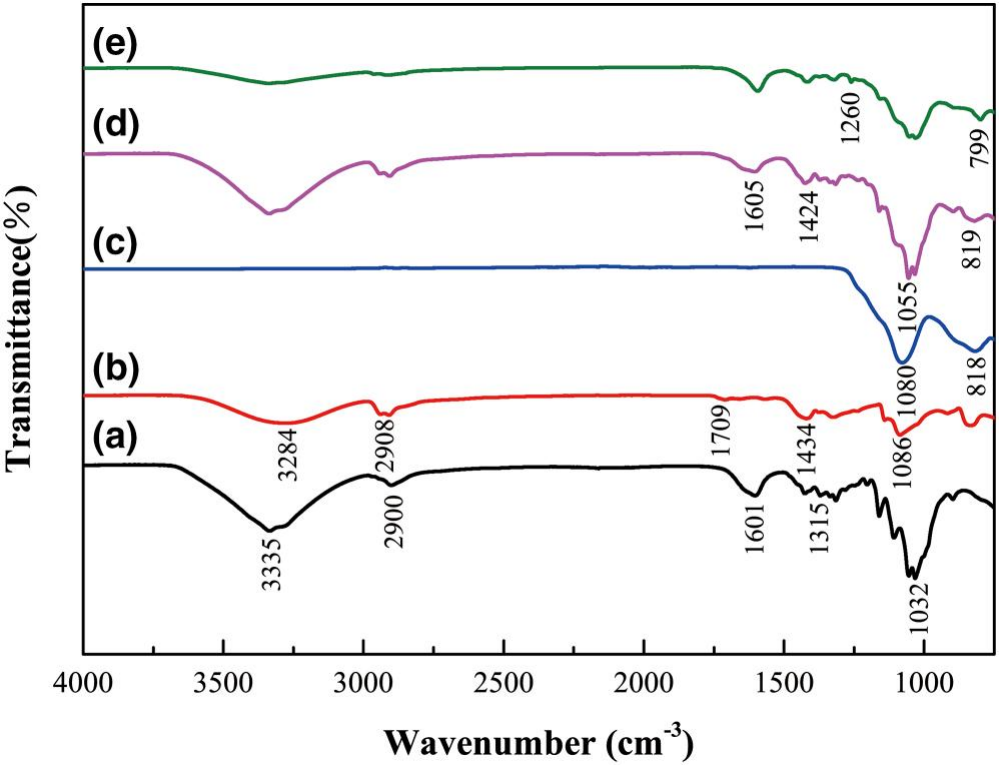
FTIR spectra. (a) CNF, (b) PVA, (c) MMT, (d) CPMA and (e) MCPMA

The spectrum of CNF (Figure 4a) showed that 3335 cm^−1^ was associated with hydroxyl group, 1601  and 1315 cm^−1^ represented the bending vibration of O–H and C–H, respectively. The peak at 2900 cm^−1^ was the tensile vibration of C–H, and 1032 cm^−1^ has a bearing on the glycosidic ring and the side group vibration, whose vicinity had many weak shoulder peaks. In the infrared spectrum of PVA, the peak value was similar to CNF, but there were still some differences (Figure 4b), the peak at 3284  and 2908 cm^−1^ were related to the –OH and –CH, respectively [29]. The peak at 1709 cm^−1^ was assigned to H–O–H bending, 1434 cm^−1^ represented C–OH bending and 1086 cm^−1^ was associated with the C–O stretching vibration absorption peak [17]. The montmorillonite exhibited a strong band at 1080 cm^−1^, which was attributed to Si–O bonds (Figure 4c). Figure 4d is the infrared spectrum of CNF/PVA/MMT composite aerogel, and the diffraction peaks of CNF, PVA, and MMT all appeared, but decreased in varying degrees. This might be due to the physical and chemical reactions among CNF, PVA and MMT. Figure 4e shows the FTIR spectrum of the silane‐modified CNF/PVA/MMT composite aerogel. Compared with the unmodified composite aerogel, the hydrophilic group was significantly weakened, and a new diffraction peak appeared at 1260 and 799 cm^−1^, which were ascribed to the characteristic vibration of asymmetric stretching of Si–O–Si and C–Si, respectively. They indicated that MTMS reacted with the composite aerogels, and polysiloxane was successfully formed on the surface of aerogels.

**FIGURE 5:**
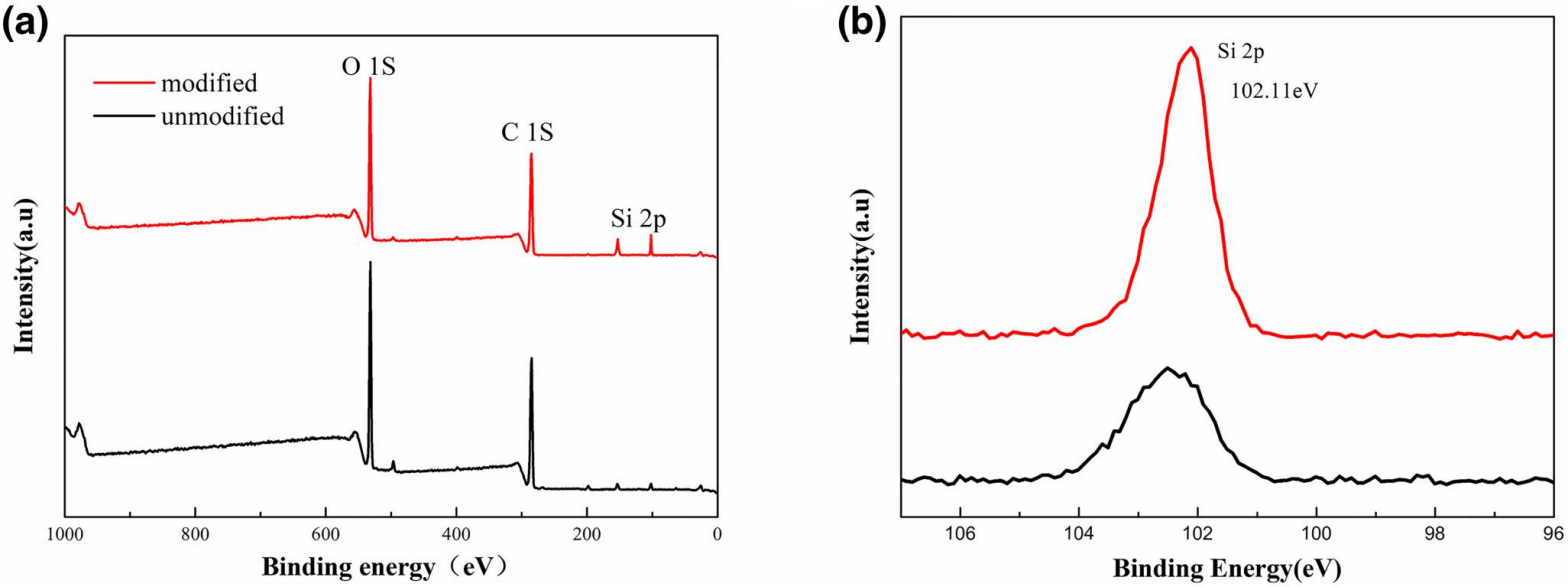
XPS comparison chart of silane‐modified aerogels and unmodified aerogels, (a) wide scan spectra and (b) narrow scan spectra of Si 2p

The interfacial bonds between MTMS and CNF/PVA substrate were further confirmed by XPS analysis, as shown in Figure 5. Compared with unmodified aerogels, two peaks at 154.08 and 102.11 eV, attributed to Si 2s and Si 2p, respectively, were intensified remarkably in the spectrum of the MTMS‐modified CNF/PVA/MMT aerogels, which suggested the presence of a large amount of silicon at the aerogel microscale. The peak of Si 2p at 102.6 eV was assigned to covalent bonds of Si–O–C, which confirmed that polysiloxane was chemically bonded onto the aerogel surface through dehydration reaction [28].

### 3.3 Mechanical properties

**FIGURE 6:**
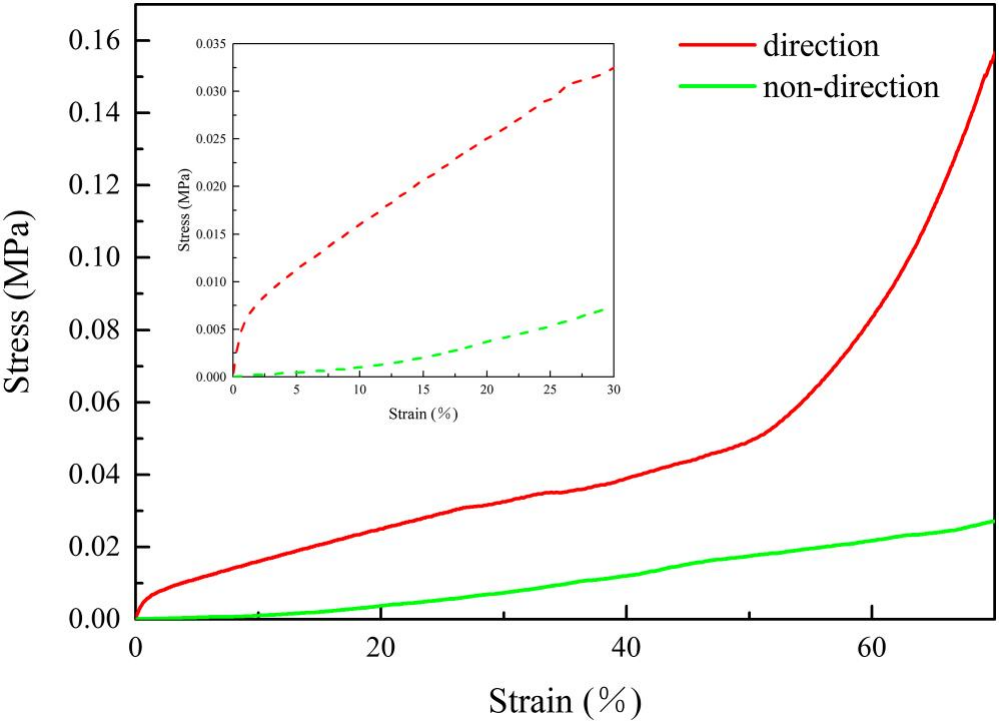
Compressive behaviour of MCPM aerogels prepared by different freeze‐drying methods

Mechanical performance has a significant role in many respects for example materials transportation and storage. Therefore, the mechanical performance is also an important feature. Many factors influenced the mechanical properties of aerogel, such as the constituent of raw materials and proportions, the macroscopic and microscopic structures of the aerogels. Take the same proportion of raw materials and components, was prepared by different preparation methods of the different structures and mechanical properties of the aerogels. Testing the compression properties of CPMA with universal mechanical testing machine. Figure 6, the curve characteristics of this characteristic deformation were consistent with previous studies. The mechanical properties of directional aerogels were better than non‐directional aerogels from the beginning. For directional aerogels, the slope of the curve was large and the rising trend was fast at the beginning of the test. Over time, the upward trend tended to be flat. However, when the strain reached 50 %, the curve rose rapidly, and the trend was stronger than the initial state. The reason for this phenomenon might be that when the sample began to compress, the pressure grew along the direction of cellulose growth due to the vertical growth of cellulose in aerogels, and the stress–strain curve rose rapidly, but the stress increased a little. As time goes on, the directional growth of cellulose fibres is gradually compressed, the stress increased with the change of aerogel morphology, and the stress–strain curve grew slowly. When the strain reached 50 %, the aerogel was compacted and the cellulose fibres were squeezed and wound together, doubling the density of the aerogel and dramatically increased its compressive strength.

Compared with other cellulose‐based aerogels described in previous studies, the compressive stress of n‐MCPMA reached 0.155 MPa at 70 % strain, which was a larger improvement [42, 43, 44, 45, 46]. The results might be caused by two aspects: on the one hand, the strong interaction between CNF and MMT, the hydrogen bond and the formation of intercalation structure. On the other hand, the combination of CNF and PVA by hydrogen bond, the close combination of CNF, PVA and MMT. Finally, the three‐dimensional network structure was formed to enhance its mechanical properties. Of course, the ultimate compressive stress of d‐MCPMA (0.155 MPa) was dozens of times higher than that of n‐MCPMA (0.025 MPa) at 70 % strain. This remarkable result was due to the directional growth of cellulose microstructure.

**FIGURE 7:**
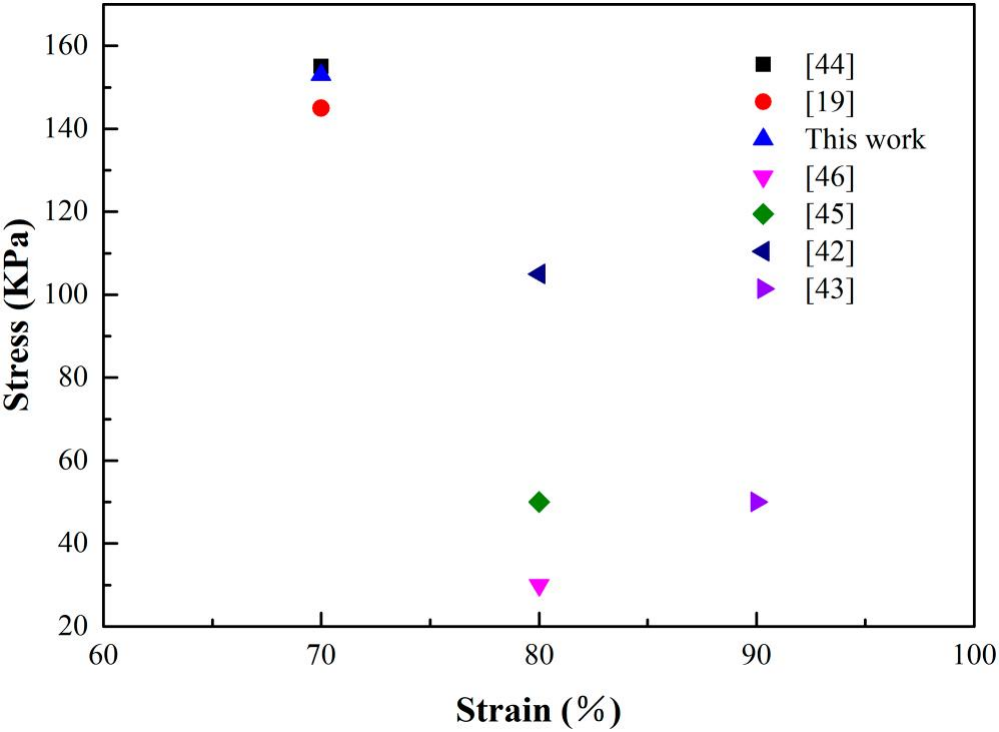
Comparison of the compressive strength of different materials

Figure 7 compares the compression strength of n‐MCPMA with other oil‐absorbing materials. The process of directional freezing causes aerogels to have an axial ordered vertical pore structure, which makes d‐MCPMA very strong in the vertical direction. However, applying pressure perpendicular to the axial direction can damage the aerogel's directional structure, making it difficult to recover. Therefore, although d‐MCPMA have high strength, its flexibility was relatively poor. But compared with other cellulosic oil‐absorbing materials, due to the directional growth of CNFs, the aerogels had directional interworking holes, which will accelerate the oil absorption rate, shorten the oil extrusion time and increase the recovery rate of aerogel, which will greatly enhance their reuse performance.

**FIGURE 8:**
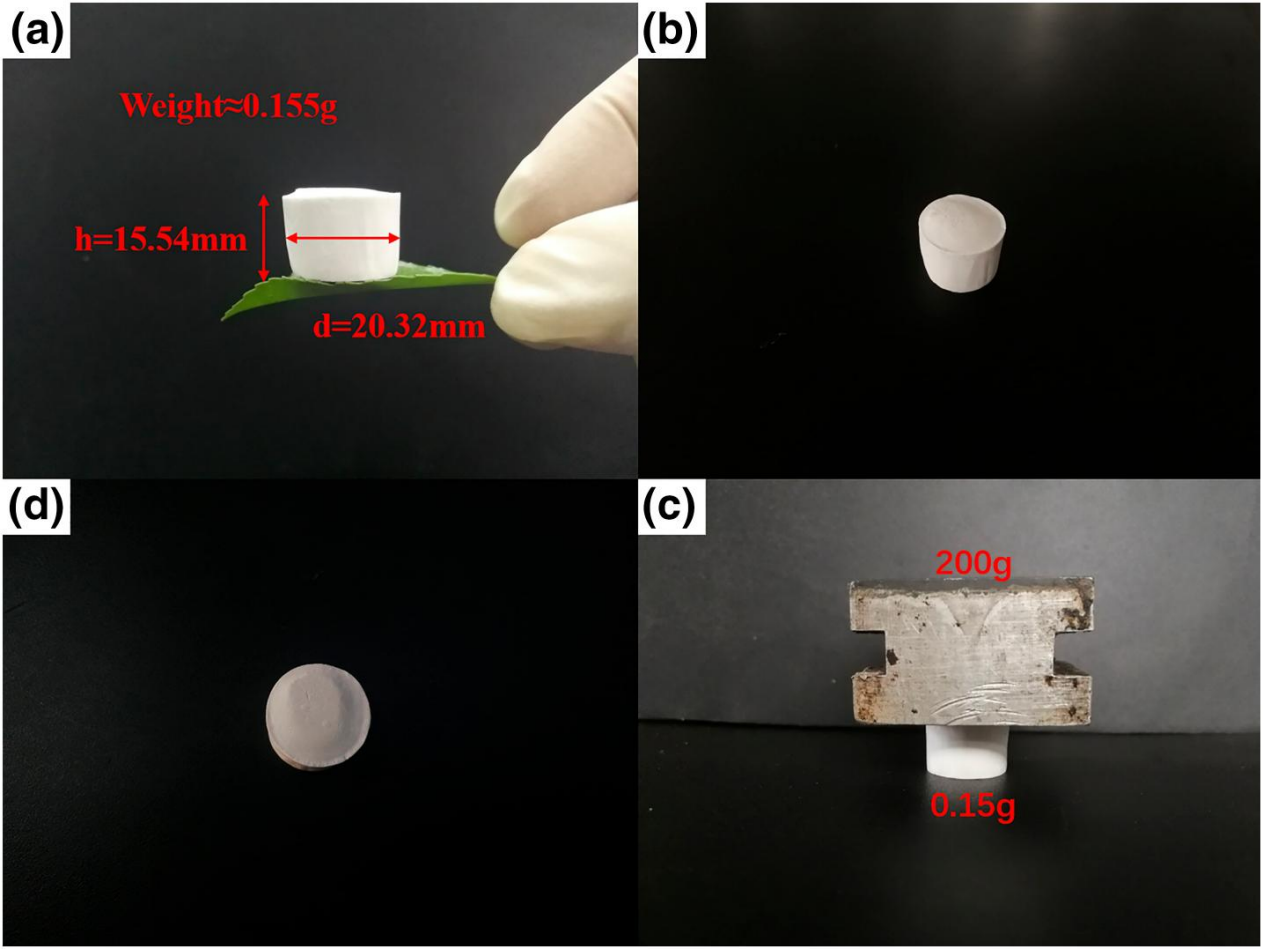
Ultra‐light cylindrical d‐CPMA supported by leaves; (a) Aerogels are placed on leaves, (b)–(d) the pre‐, middle and later performances of the d‐CPMA supporting a 200 g mass load

To show the characteristics of aerogels lightweight and high strength more directly, we made a small experiment in Figure 8.

The CPMA was selected at random, the height is 15.54 mm, the diameter is 20.32 mm and the weight is 0.155 g. We put the aerogel on the surface of the leaves, the aerogel did not drop, and the leave did not droop. Then we put a 200 g piece of iron on top of the aerogel (bearing more than 1290 times its weight). After 48 h of loading, the height of aerogel did not change, and after 2 weeks, only a little indentation appeared on the contact surface. This high strength was mainly attributed to the combination of CNF, PVA and MMT, as well as the directional freeze‐drying microporous structure. It can ensure that the properties of aerogels will not change during storage and transportation.

### 3.4 Surface wettability

**FIGURE 9:**
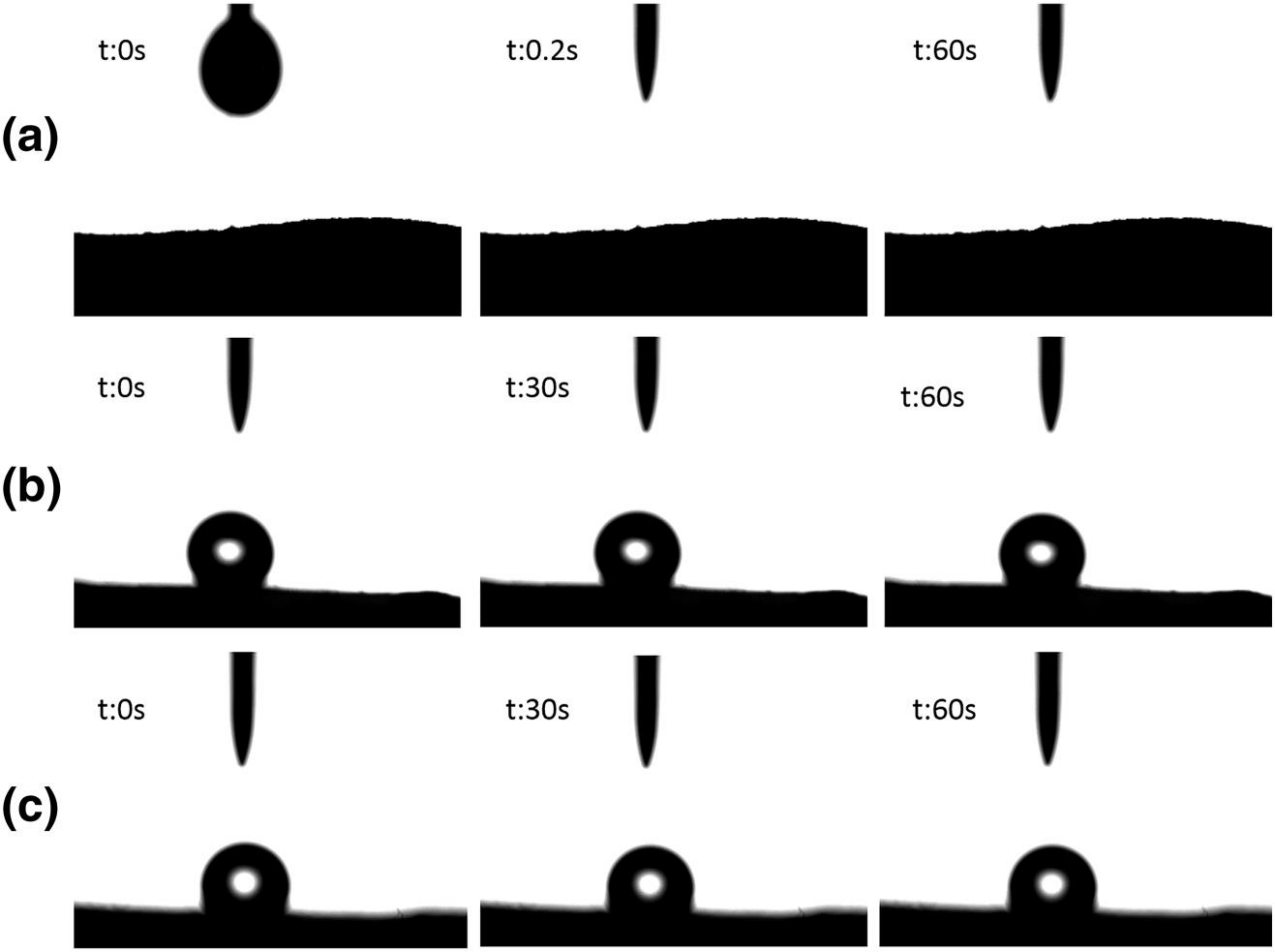
Water contact angle of (a) CPMA, (b) MCPMA and (c) MCA

Hydrophobicity is one of the important standards to measure the properties of oil‐absorbing materials. The surface wettability of modified CNF aerogel (MCA), CPMA, MCPMA (studies have shown that freezing method has little effect on aerogel hydrophobicity [19]) was investigated by contact angle measurement. Because of the large number of hydroxyl groups in the molecular chains of CNF and PVA, the CPMA exhibited strong hydrophilicity. As shown in Figure 9a, when 2 μL H_2_O came into contact with the unmodified CPMA, the water droplets were immediately absorbed in 0.2 s, and the WCA of 0°. After being treated with MTMS, the WCA of MCPMA and MCA was as high as 140° and 128° (Figure 9b, 9c). After 60 s, the water droplets still existed on the aerogel surface, and the WCA not significantly changed. CPMA was an inter‐microporous structure that promoted silane modifier into aerogel, so that aerogels were silaned completely and hydrophobicity reached the maximum. These results showed that the aerogel had a highly hydrophobic surface after silane treatment.

### 3.5 Hydrophobic lipophilic and absorption capacity

**FIGURE 10:**
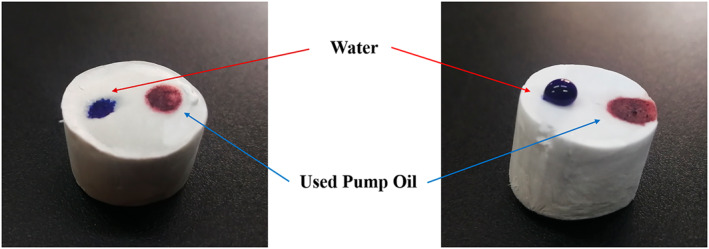
Hydrophobic and lipophilic of (a) d‐CPMA and (b) d‐MCPMA

Silane‐modified CPMA is one of the ideal materials for oil and water treatment because of its excellent lipophilicity and hydrophobicity ability, as well as its porous structure. Figure 10 shows that the MCPMA had a good hydrophobic and oil‐wet capability compared with the unmodified aerogel. Water was stained with Methylene Blue, and pump oil was stained with Sudan Ⅲ.

**FIGURE 11:**
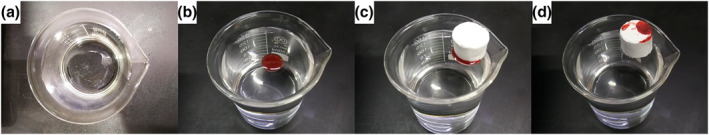
Removal of diesel oil (dyed with Sudan Ⅲ) from the water surface using MCPMA

**FIGURE 12:**
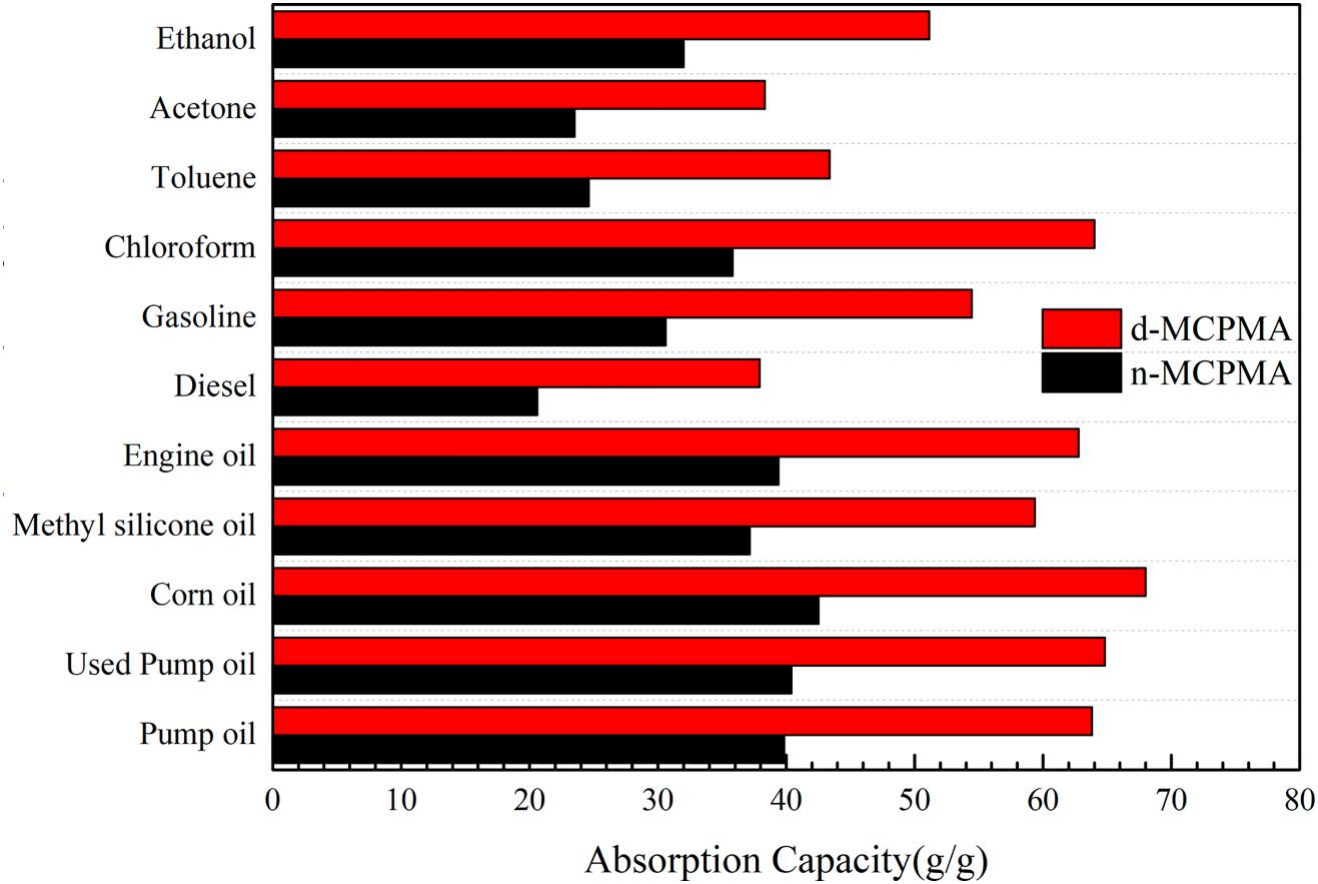
Absorption capacity of MCPGA (d‐MCPMA, n‐MCPMA) for different oils and organic liquids

When the water and oil droplets came into contact with the CPMA, the water and oil were absorbed quickly and a sag appeared at the point where the liquid drop disappeared. However, after modification, the situation changed. The water droplets remained on the surface of the aerogel, and the oil droplets were absorbed by the aerogel. Figure 11a–11d shows the process of removed diesel (stained with Sudan Red) from the water surface by MCPMA, the oil and aerogel were hydrophobic. When the aerogel was in contact with oil, the oil was absorbed completely and the water would become clean again, and the aerogel was far from reaching their adsorption limits. Different kinds of oils and organic solvents were used, and the weight of aerogels before and after oil absorption was determined. Last, the adsorption rate of aerogels was obtained. Figure 12 shows the absorption capacity of the MCPMA (d‐MCPMA, n‐MCPMA) for different oils and organic liquids. The oils and organic liquids tested included ethanol, acetone, toluene, chloroform, gasoline, diesel, engine oil, methyl silicone oil, corn oil, used pump oil and pump oil.

The adsorption capacity of aerogels is different for different kinds of oils and organic solvents. Figure 12 shows the absorption capacity of d‐MCPMA for oils and the organic solvents were higher, 38–68 times and 40–64 times, respectively. However, the n‐MCPMA was relatively low, only 20–41 times and 24–36 times. Maybe it was because of the high density and viscosity, oil was mainly stored in the pores of aerogels, and the adsorption of organic solvent mainly depended on the surface tension and the density of organic solvent. As is known to all, capillary force was the main factor in aerogel adsorption, due to the capillary force between the liquid surface and the aerogel holes and channels, the liquid could spontaneously enter the aerogel interior. MCPMA was a porous material, d‐MCPMA had a more stable pore structure, more uniform size and distribution than n‐MCPMA, so it could absorb more oil and organic solvents. Besides, the ability of the d‐MCPMA to absorb oil was much better than that of other materials (Table 2).

**TABLE 2:** Comparison of adsorption properties (oil) and hydrophobicity of different materials

Absorbent materials	WCA(°)	Capacity (g/g)	Ref
CNFs	144	44	[47]
Carbon aerogel	140	14‐26	[48]
NFC/PVA/SAC	159	35‐85	[49]
Cotton cellulose aerogel	154	20‐42	[50]
Chitosan/Cellulose aerogel	152	14‐28	[51]
Sodium alginate/CNF	148	34	[52]
Graphene/cellulose/silica	157	39‐68	[53]
NFC/OA/Fe_3_O_4_	84	68	[54]
TMCs/CNT/CMC	148	28	[55]
Poly(lactic acid) foam	151	32	[56]
Bamboo fibre aerogel	132	7‐11	[57]
Cellulose/montmorillonite	No	4‐7	[40]
CNF/PVA/MMT	140	40‐68	This work

**FIGURE 13:**
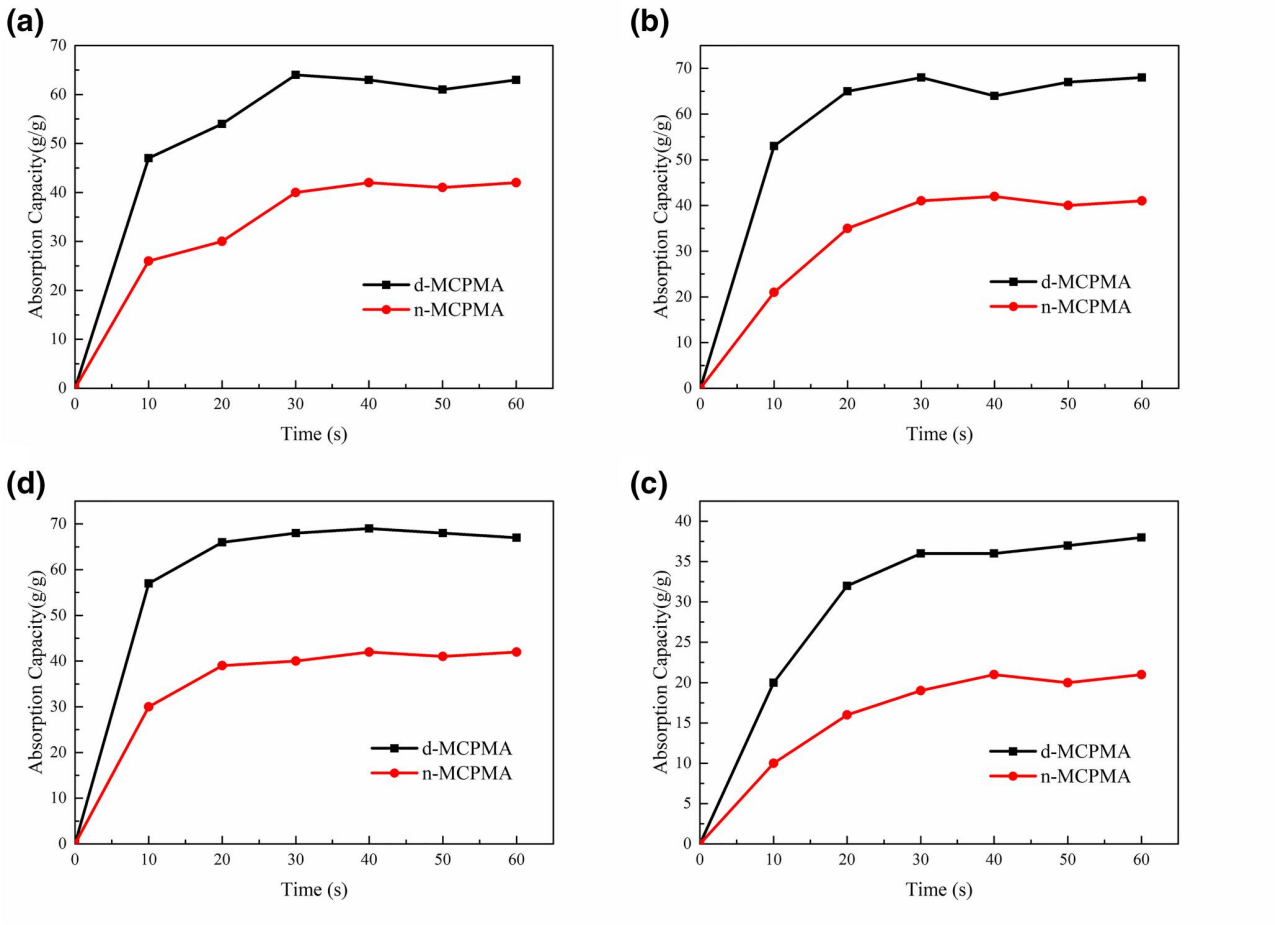
The absorption capacity of d‐MCPMA, n‐MCPMA) to adsorb (a) pump oil; (b) engine oil; (c) diesel oil and (d) corn oil as a function of time

As we all know, the higher the viscosity of the liquid, the slower the molecular movement and the worse the fluidity of the liquid. As a result, the performance of the liquid was different in the adsorption, filtration and other behaviours. In Figure 13, CPMA takes 30–40 s for silicone oil, engine oil and diesel oil to reach the adsorption limit, whereas it only takes 20 s for corn oil. Viscosity ratio of four oils: pump oil > diesel oil > engine oil > corn oil. In the adsorption process of four kinds of oil, the initial adsorption speed was the fastest, but with the advance of the adsorption process, the adsorption speed gradually slowed down, and finally reached the adsorption limit, the speed remains unchanged. This might be due to the unique three‐dimensional porous interpenetrating network structure of aerogels provided a larger number of adsorption sites, but as time progressively, the adsorption sites were gradually occupied by oil, resulting in a slow absorption rate. Finally, when all adsorption sites and spaces were completed, the adsorption value reached saturation, and the absorption was suspended. It could be seen from Figure 13b and 13c that the adsorption rate of n‐MCPMA was slower than that of d‐MCPMA, which might be due to the disordered arrangement of pore structure, resulting in the uneven oil absorption process and the slow adsorption rate.

## 4. CONCLUSION

Herein, a new, green simple liquid nitrogen directed freezing method was used to prepare low density (25.62 mg⋅cm^−3^), high porosity (96.1 %) CNF/PVA/MMT aerogels, and the shrinkage of composite aerogel was less than 6.0 % and the morphology structure was stable after freeze‐dried. Compared with cellulosic aerogels, CPMA has excellent mechanical properties, with the compressive stress at 70% strain reaching 0.15 MPa. And it can carry heavy objects weighing more than 1290 times its weight for up to 2 weeks without changing its shape, which is convenient for transportation and storage. After the hydrophobic modification with MTMS, the WCA of the composite aerogel can reach 140°, which has a strong hydrophobic ability. The ultimate adsorption capacity of aerogel is 68 times its initial weight, and it can be adsorbed within 30 s, which is far better than cellulosic aerogel, and the adsorption speed is relatively fast. Compared with mineral‐based adsorption materials, after the adsorption is completed, the MCPMA can be simply recovered from the water surface, reused or incinerated without causing secondary pollution. MCPMA combines the advantages of both CNFs and montmorillonite to make up for the shortcomings between the two. It is an easy‐to‐preparation, environmentally friendly and excellent performance oil‐absorbing material, which provides a new research approach for our wastewater treatment.

## References

[1] Yang, Y. , Deng, Y, H. , Tong, Z. , et al.: Multifunctional foams derived from poly (melamine formaldehyde) as recyclable oil absorbents. J. Mater. Chem. 2(26), 9994–9999 (2014)

[2] Gong, X, Y. , Yi, X, W. , Hong, B, Z. , et al.: Highly porous, hydrophobic, and compressible cellulose nanocrystals/PVA aerogels as recyclable absorbents for oil‐water separation. ACS Sustainable Chem. Eng. 7(13), 11118–11128 (2019)

[3] Barbier, E, B. , Moreno‐Mateos, D. , Rogers, A, D. , et al.: Ecology: protect the deep sea. Nature. 505(7484), 475–477 (2014) 24459714 10.1038/505475a

[4] Schrope, M. : Oil spill: deep wounds. Nature. 472(7342), 152–154 (2011) 21490648 10.1038/472152a

[5] Zhang, J, P. , Seeger, S. : Superhyrophobic materials: polyester materials with superwetting silicone nanofilaments for oil/water separation and selective oil absorption. Adv. Funct. Mater. 21(24), 4699–4704 (2015)

[6] Zhao, Y, F. , Zhong, K. , Liu, W. , et al.: Preparation and oil adsorption properties of hydrophobic microcrystalline cellulose aerogel. Cellulose. 27, 7663–7675 (2020)

[7] Nwadiogbu, J, O. , Ajiwe, V, I, E. , Okoye, P, A, C. : Removal of crude oil from aqueous medium by sorption on hydrophobic corncobs: equilibrium and kinetic studies. J. Taibah Univ. Sci. 10, 56–63 (2016)

[8] Fard, A, K. , Manawi, G. , Manawi, Y. , et al.: Outstanding adsorption performance of high aspect ratio and superhydrophobic carbon nanotubes for oil removal. Chemosphere. 116, 142–155 (2016) 10.1016/j.chemosphere.2016.08.099 27588573

[9] Araby, S. , Qiu, A. , Wang, R, Y. , et al.: Aerogels based on carbon nanomaterials. J. Mater. Sci. 51(20), 9157–9189 (2016)

[10] He, J. , Zhao, H, Y. , Li, X, L. , et al.: Superelastic and superhydrophobic bacterial cellulose/silica aerogels with hierarchical cellular structure for oil absorption and recovery. J. Hazard. Mater. 346, 199–207 (2018) 29275109 10.1016/j.jhazmat.2017.12.045

[11] Nguyen, S, T. , Feng, J, D. , Wong, J, P. , et al.: Advanced thermal insulation and absorption properties of recycled cellulose aerogels. Colloid Surface Physicochem. Eng. Aspect. 445, 128–134 (2014)

[12] Yang, W. , Wang, N, N. , Ping, P. , et al.: A novel 3D network architectured hybrid aerogel comprising epoxy, graphene and hydroxylated boron nitride nanosheets. ACS Appl. Mater. Interfaces. 10, 40032–40043 (2018) 30379530 10.1021/acsami.8b15301

[13] Hwang, D, W. , Park, J, B. , Sung, D, C. , et al.: 3D graphene‐cellulose nanofiber hybrid scaffolds for cortical reconstruction in brain injuries. 2D Mater., 6, 1–37 (2019)

[14] Shefa, A, A. , Amirian, J. , Kang, H, J. , et al.: In vitro, and, in vivo, evaluation of effectiveness of a novel TEMPO‐oxidized cellulose nanofiber‐silk fibroin scaffold in wound healing. Carbohydr. Polym. 177, 284–296 (2017) 28962770 10.1016/j.carbpol.2017.08.130

[15] Miao, X, R. , Lin, J, Y. , Bian, F, G. : Utilization of discarded crop straw to produce cellulose nanofibrils and their assemblies. J. Bioresour. Bioprod. 3, 1–11 (2020)

[16] Feng, J, D. , Nguyen, S, T. , Fan, Z. , et al.: Advanced fabrication and oil absorption properties of super‐hydrophobic recycled cellulose aerogels. Chem. Eng. J. 270, 168–175 (2015)

[17] Jin, C, D. , Han, S, J. , Li, J, P. , et al.: Fabrication of cellulose‐based aerogels from waste newspaper without any pretreatment and their use for absorbents. Carbohydr. Polym. 123, 150–156 (2015) 25843846 10.1016/j.carbpol.2015.01.056

[18] Mi, H, Y. , Jing, X. , Politowicz, A, L. , et al.: Highly compressible ultra‐light anisotropic cellulose/graphene aerogel fabricated by bidirectional freeze drying for selective oil absorption. Carbon. 12869, 1–32 (2018)

[19] Zhou, L, J. , Zhai, S, C. , Chen, Y, M. , et al.: Anisotropic cellulose nanofibers/polyvinyl alcohol/graphene aerogels fabricated by directional freeze‐drying as effective oil adsorbents. Polymers. 11, 712–726 (2019) 10.3390/polym11040712 PMC6523222 31003569

[20] Zhang, Z. , Sebe, G. , Rentsch, D. , et al.: Ultralightweight and flexible Silylated nanocellulose sponges for the selective removal of oil from water. Chem. Mater. 26(8), 2659–2668 (2014)

[21] Hsieh, H, T. , Chang, H, M. , Lin, W, J. , et al.: Poly‐methyl methacrylate/polyvinyl alcohol copolymer agents applied on diabetic wound dressing. Sci. Rep. 7(1), 9531–9540 (2017) 28842691 10.1038/s41598-017-10193-5 PMC5572720

[22] Dai, H. , Huang, Y. , Huang, H, H. : Eco‐friendly polyvinyl alcohol/carboxymethyl cellulose hydrogels reinforced with graphene oxide and bentonite for enhanced adsorption of methylene blue. Carbohydr. Polym. 185, 1–11 (2018) 29421044 10.1016/j.carbpol.2017.12.073

[23] Pan, Y, X. , Liu, Z. , Wang, W, C. , et al.: Highly efficient macroporous adsorbents for toxic metal ions in water system based on polyvinyl alcohol‐formaldehyde sponges. J. Mater. Chem. 4(7), 2537–2549 (2016)

[24] Liu, C, T. , Shao, Z, Q. , Wang, J, Q. , et al.: “Eco‐friendly polyvinyl alcohol/cellulose nanofifiber–Li+ composite separator for high performance lithium‐ion batteries”. RSC Adv. 6, 97912–97920 (2016)

[25] Niazi, M, B, K. , Jahan, Z. , Berg, S, S. , et al.: Mechanical, thermal and swelling properties of phosphorylated nanocellulose fibrils/PVA nanocomposite membranes. Carbohydr. Polym. 177, 258–268 (2017) 28962767 10.1016/j.carbpol.2017.08.125

[26] Cazón, P. , Vázquez, M. , Velazquez, G. : Cellulose‐glycerol‐polyvinyl alcohol composite films for food packaging: evaluation of water adsorption, mechanical properties, light‐barrier properties and transparency. Carbohydr. Polym. 195, 432–443 (2018) 29804996 10.1016/j.carbpol.2018.04.120

[27] Xu, Z, Y. , Jiang, X, D. , Zhou, H. , et al.: Preparation of magnetic hydrophobic polyvinyl alcohol (PVA)–cellulose nanofiber (CNF) aerogels as effective oil absorbents. Cellulose. 25, 1217–1227 (2018)

[28] Zhang, X, X. , Wang, H, K. , Cai, Z, Y. , et al.: “Highly compressible and hydrophobic anisotropic aerogels for selective oil/organic solvent absorption”, ACS Sustainable Chem. Eng., 2019, 7, pp 332−340

[29] Zhou, T. , Cheng, X, D. , Pan, Y, L. , et al.: “Mechanical performance and thermal stability of polyvinyl alcohol–cellulose aerogels by freeze drying”. Cellulose. 26, 1747–1755 (2019)

[30] Zhai, T, L. , Zheng, Q, F. , Cai, Z, Y. , et al.: Synthesis of polyvinyl alcohol/cellulose nanofibril hybrid aerogel microspheres and their use as oil/solvent superabsorbents. Carbohydr. Polym. 148, 300–308 (2016). 27185143 10.1016/j.carbpol.2016.04.065

[31] Fan, L, H. , Lu, Y, Q. , Yang, L, Y. , et al.: Fabrication of polyethylenimine‐functionalized sodium alginate/cellulose nanocrystal/polyvinyl alcohol core–shell microspheres ((PVA/SA/CNC)@PEI) for diclofenac sodium adsorption. J. Colloid Interface Sci. 554, 48–58 (2019) 31279272 10.1016/j.jcis.2019.06.099

[32] Li, L, F. , Wang, L. , Liu, S. , et al.: Effect of water state and polymer chain motion on the mechanical properties of a bacterial cellulose and polyvinyl alcohol (BC/PVA) hydrogel. RSC Adv. 5, 25525–25531 (2015)

[33] Uddin, F. : Clays, Nanoclays, and Montmorillonite Minerals. Metall. Mater. Trans. A. 39(12), 2804–2814 (2008)

[34] Anirudhan, T, S. , Ramachandran, M. : Adsorptive removal of basic dyes from aqueous solutions by surfactant modified bentonite clay (organoclay): kinetic and competitive adsorption isotherm. Process Safety Environ. Protect. 95, 215–225 (2015)

[35] Islam, M, S. , Rahaman, M, S. , Yeum, J, H. : Electrospun novel super‐absorbent based on polysaccharide–polyvinyl alcohol–montmorillonite clay nanocomposites. Carbohydr. Polym. 115, 69–77 (2015) 25439870 10.1016/j.carbpol.2014.08.086

[36] Madyana, O, A. , Fan, M, Z. : Organic functionalization of clay aerogel and its composites through in‐situ crosslinking. Appl. Clay Sci. 168, 374–381 (2019)

[37] Liu, A, D. , Medina, L. , Berglund, L, A. : High‐strength nanocomposite aerogels of Ternary composition: poly (vinyl alcohol), clay, and cellulose nanofibrils. ACS Appl. Mater. Interfaces. 9(7), 6453–6461 (2017) 28155270 10.1021/acsami.6b15561

[38] Alhwaige, A, A. , Ishida, H. , Qutubuddin, S. : Carbon aerogels with excellent CO2 adsorption capacity synthesized from clay‐reinforced Biobased chitosan‐polybenzoxazine nanocomposites. ACS Sustainable Chem. Eng. 4, 1286–1295 (2016)

[39] Frindy, S. , Primo, A. , Qaiss, A, E, K. , et al.: Insightful understanding of the role of clay topology on the stability of biomimetic hybrid chitosan‐clay thin films and CO2‐dried porous aerogel microspheres. Carbohydr. Polym. 4, 2601–2605 (2016) 10.1016/j.carbpol.2016.03.077 27112884

[40] Long, L, Y. , Li, F, F. , Weng, Y, X. , et al.: Effects of sodium montmorillonite on the preparation and properties of cellulose aerogels. Polymers. 11, 415–424 (2019) 10.3390/polym11030415 PMC6473606 30960399

[41] Chen, W, S. , Yu, H, P. , Liu, Y, X. : Preparation of millimeter‐long cellulose I nanofibers with diameters of 30‐80 nm from bamboo fibers. Carbohydr. Polym. 86, 453–461 (2011)

[42] Javadi, A. , Zheng, Q, F. , Payen, F. , et al.: Polyvinyl alcohol‐cellulose nanofibrils‐graphene oxide hybrid organic aerogels. ACS Appl. Mater. Interfaces. 5(13), 5969–5975 (2013) 23789837 10.1021/am400171y

[43] Yang, X. , Cranston, E, D. : Chemically cross‐linked cellulose nanocrystal aerogels with shape recovery and superabsorbent properties Chem. Mater. 26(20), 6016–6025 (2014)

[44] Zhou, S, K. , Wang, M. , Chen, X. , et al.: Facile template synthesis of microfibrillated cellulose/polypyrrole/silver nanoparticles hybrid aerogels with electrical conductive and pressure responsive properties. ACS Sustainable Chem. Eng. 3(12), 3346–3354 (2015)

[45] Wicklein, B. , Kocjan, A. , Salazar‐Alvarez, G. , et al.: Thermally insulating and fire‐retardant lightweight anisotropic foams based on nanocellulose and graphene oxide. Nat. Nanotechnol., 277–283 (2015) 25362476 10.1038/nnano.2014.248

[46] Jiang, F. , Hsieh, Y. : Amphiphilic superabsorbent cellulose nanofibril aerogels. J. Mater. Chem. A. 2(18), 6337–6342 (2014)

[47] Mulyadi, A. , Zhang, Z. , Deng, Y, L. : Fluorine‐free oil absorbents made from cellulose nanofibril aerogels. ACS Appl. Mater. Interfaces. 8, 2732–2740 (2016) 26761377 10.1021/acsami.5b10985

[48] Lei, E. , Wei, L. , Chunhui, M. , et al.: An ultra‐lightweight recyclable carbon aerogel from bleached softwood kraft pulp for efficient oil and organic absorption. Mater. Chem. Phys. 214, 291–296 (2018)

[49] Chhajeda, M. , Yadava, C. , Agrawal, A, K. , et al.: Esterified superhydrophobic nanofibrillated cellulose based aerogel for oil spill treatment. Carbohydr. Polym. 226, 115286 (2019) 31582050 10.1016/j.carbpol.2019.115286

[50] Wang, J, T. , Liu, S, Y. : Remodeling of raw cotton fiber into flexible, squeezing‐resistant macroporous cellulose aerogel with high oil retention capability for oil/water separation. Separ. Purif. Technol. 221, 303–310 (2019)

[51] Li, Z, Y. , Shao, L. , Hu, W, B. , et al.: Excellent reusable chitosan/cellulose aerogel as an oil and organic solvent absorbent. Carbohydr. Polym. 191, 183–190 (2018) 29661308 10.1016/j.carbpol.2018.03.027

[52] Yang, J. , Xia, Y, F. , Xu, P. , et al.: Super‐elastic and highly hydrophobic/superoleophilic sodium alginate/cellulose aerogel for oil/water separation. Cellulose. 25, 3533–3544 (2018)

[53] Mi, H, Y. , Jing, X. , Huang, H, X. , et al.: Superhydrophobic graphene/cellulose/silica aerogel with hierarchical structure as superabsorbers for high efficiency selective oil absorption and recovery. Ind. Eng. Chem. Res. 57, 1745–1755 (2018)

[54] Gu, H, B. , Zhou, X, M. , Lyu, S, Y. , et al.: Magnetic nanocellulose‐magnetite aerogel for easy oil adsorption. J. Colloid Interface Sci. 560, 849–856 (2018) 10.1016/j.jcis.2019.10.084 31708258

[55] Parmar, K, R. , Dora, D, T, K. , Pant, K, K. , et al.: An ultra‐light flexible aerogel‐based on methane derived CNTs as a reinforcing agent in silica‐CMC matrix for efficient oil adsorption. J. Hazard. Mater. 375, 206–215 (2019) 31071618 10.1016/j.jhazmat.2019.04.017

[56] Wang, X, L. , Pan, Y, M. , Liu, X, H. , et al.: Facile fabrication of superhydrophobic and eco‐friendly polylactic acid foam for oil‐water separation via skin‐peeling. ACS Appl. Mater. Interfaces. 11, 14352–14367 (2019) 10.1021/acsami.9b02285 30916921

[57] Nguyen, D, D. , Vu, C, M. , Vu, H, T. , et al.: Micron‐size white bamboo fibril‐based silane cellulose aerogel: fabrication and oil absorbent characteristics. Materials. 12, 1407–1021 (2019) 10.3390/ma12091407 PMC6539521 31052179

